# Generating a Full Cycle of Alternative Current Using a Triboelectric Nanogenerator for Energy Harvesting

**DOI:** 10.3390/mi16010011

**Published:** 2024-12-25

**Authors:** Aso Ali Abdalmohammed Shateri, Fengling Zhuo, Nazifi Sani Shuaibu, Rui Wan, Liangquan Xu, Dinku Hazarika, Bikash Gyawali, Xiaozhi Wang

**Affiliations:** 1 College of Information Science and Electronic Engineering, Zhejiang University, Hangzhou 310027, China; asoali202@zju.edu.cn (A.A.A.S.); fenglingzhuo@zju.edu.cn (F.Z.); zuhudu@zju.edu.cn (N.S.S.); wanrui@zju.edu.cn (R.W.); xuliangquan@zju.edu.cn (L.X.); hazarikadink@zju.edu.cn (D.H.); 2 Physics Department, College of Education, University of Garmian, Kalar 46021, Iraq; 3 College of Electrical Engineering, Zhejiang University, Hangzhou 310058, China; bikash_gyawali@zju.edu.cn

**Keywords:** triboelectric nanogenerators, gear system, energy harvester, freestanding rotary triboelectric nanogenerators, alternative current (AC)

## Abstract

The triboelectric nanogenerator (TENG) has emerged as a promising technology for efficiently converting ambient mechanical energy into electrical energy. Among various designs, the disk-based rotational TENG has demonstrated significant potential, as it can continuously harvest energy in a sliding mode via a grating mechanism. However, horizontal mechanical energy is more common than rotational energy in many practical applications. Herein, the present study introduces a novel device: the double horizontal linear-to-rotational triboelectric nanogenerator (DHLR-TENG). This innovative approach utilizes a gear system to convert horizontal linear mechanical energy into electrical energy. The experimental results revealed that the DHLR-TENG produces a full cycle of alternating current (AC) when integrated into an electrical circuit. It consistently delivers robust performance with an open-circuit voltage of 544 V, a short-circuit current of 61.16 µA, and a maximum power output of 33.27 mW. Additionally, the device durability, capable of withstanding over 1,000,000 cycles, makes it highly effective for powering small electronic devices, such as charging capacitors and illuminating commercial LEDs. The DHLR-TENG’s versatility and efficiency mark it as a major advancement in energy harvesting, with broad implications for powering portable electronic devices in a wide range of environments.

## 1. Introduction

The triboelectric nanogenerator (TENG) has gained significant attention in the last few years as a potential substitute for conventional energy-harvesting mechanisms. The TENG device is capable of converting mechanical energy from the surrounding environment into electrical energy through the processes of contact electrification and electrostatic induction [1,2,3]. The TENG has gained significant interest as a next-generation technology that surpasses conventional mechanical energy-harvesting technology, owing to its straightforward design process, environmentally friendly functioning, and extensive range of available materials [4,5,6].

The functional mechanism of the TENG relies on the combination of contact electrification and electrostatic induction. Initially, when two items with distinct positions in the triboelectric series come into contact, a charge transfer process induces positive and negative charges on the surfaces of both objects [7,8,9]. When the two things are placed at a certain distance from each other, an electric field is generated between them. In order to attain electrical balance, the transfer of electrons occurs through electrodes that are affixed to the rear surfaces of the two objects [4,10].

The TENG variant that operates in a sliding mode has garnered significant attention in academic research because of its potential for achieving superior electrical functionality. This is attributed to the fact that when two objects slide against each other, as opposed to simply making contact, a greater amount of charge is transmitted between their surfaces [7,11,12]. A disk-based TENG was developed as an illustrative case to capture rotating mechanical energy [11,13,14,15], demonstrating exceptional electrical characteristics. The disk-based TENG was operated through the rotational motion of two coaxial circular disks. When the two disks make contact, electrostatic induction takes place due to a rotary–sliding motion, resulting in the transfer of electrons between two electrodes on the surface of stators that have complimentary patterns. In contrast to a contact separation mode TENG that operates by linear force, the disk-based TENG operating in sliding mode and driven by rotational force offers distinct advantages in terms of its electrical characteristics [2,10,16,17,18]. For instance, the continuous and steady generation of output power is a characteristic of the TENG under consideration. In contrast, the contact separation TENG exhibits a brief but intense burst of output power, which may pose challenges in terms of power management circuit design. Nevertheless, it should be noted that disk-based TENG is restricted in its operation to a sole rotating force. The availability of rotational mechanical energy is comparatively lower than that of linear mechanical energy [2]. A limited number of researchers have documented the existence of disk-based TENGs that operate in a sliding mode, utilizing linear mechanical force derived solely from fluid-like sources such as wind and water [4,19,20,21,22]. Furthermore, it has been demonstrated that a fan plays a crucial role in the conversion of linear force to rotating force [2,3,7]. In this study, we utilize the force conversion mechanism to enhance the application potential of the TENG.

This study introduces a new disk-based TENG that effectively captures ample linear mechanical energy from machine movements while also having the capability to harness energy from human movement. A pair of double disks made of identical materials are prepared for the purpose of providing a complete alternative current (AC) output. A double horizontal-to-rotational triboelectric nanogenerator (DHLR-TENG) is successfully built as an experimental device, with the ability to transform horizontal force into rotational force. A gear system that is appropriately built facilitates the conversion of horizontal mechanical force into rotating mechanical force. In this system, where copper (Cu) slides on PTFE, it has been observed that velocity-strengthening and -weakening phenomena occur due to different sliding velocities in the system. Meanwhile, this work reveals the remarkable durability of the TENG system. Achieving an impressive reliably when operating for more than 1,000,000 cycles, the system highlights significant advancements in optimizing performance and energy conversion and ensures long-term reliability and stability. The generation of a complete alternating current (AC) cycle in this study ensures maximum energy-harvesting efficiency and stability in the rectified direct current (DC) output, addressing fluctuations commonly observed in incomplete AC systems and enhancing the system’s practicality for diverse applications. This extensive durability makes the TENG system promising for practical applications, offering both effective energy conversion and sustained operation over extended periods, making it a viable solution for various energy-harvesting needs. As a result, the DHLR-TENG has the capability to supply electrical energy to compact electronic components such as capacitors and light-emitting diodes (LEDs).

## 2. Experimental Section

### 2.1. Fabrication of the DHLR-TENG

#### 2.1.1. Design and Fabrication of the Disk (Stator and Rotator)

Initially, using the SolidWorks software **(Version 34.1)**, the rotator and stator were designed and then sent to the factory for fabrication. Copper (Cu) and polytetrafluoroethylene (PTFE) are widely used as positive and negative materials in triboelectric nanogenerators (TENGs) due to their complementary triboelectric properties and favorable material characteristics. Copper, being high in the triboelectric series, tends to lose electrons and become positively charged, while PTFE, positioned low in the series, gains electrons and becomes negatively charged. This significant charge transfer is crucial for generating a high output voltage. Copper’s excellent electrical conductivity, mechanical properties, and durability, combined with PTFE’s high electron affinity, chemical stability, and low friction coefficient, make them ideal for TENG applications. Additionally, their ease of fabrication and cost-effectiveness support large-scale production. This combination is particularly effective for energy-harvesting and wearable electronics, where reliable and consistent energy generation is essential [23]. The substrate was green glass epoxy with a thickness of 1.5 mm and diameters of 150 mm for the rotator and 160 mm for the stator. The substrate consisted of a hole with a diameter of 5 mm formed in the center of the substrate for rotation along a fixed axis to transfer energy. A hole with a radius of 7.5 mm was also formed in the center of the stator substrate. Also, the protruding patterns consisted of Cu. The radius of the rotator and stator was 75 mm, and the thickness was 1.5 mm. Nineteen radially arrayed sectors of Cu were patterned onto the surface of the rotator, and thirty-eight of these sectors were patterned onto the surface of the stator. Each sector unit had a central angle of 9.47 degrees and an outermost length of 55 mm, with the outermost spacing lengths at 11.15 mm on the rotator and 1 mm on the stator, as shown in the enlarged surface images in Supplementary Figure S2. The patterned Cu on the stator was divided into electrode 1 and electrode 2. The sectors on the stator were divided into 19 sectors for electrode 1 and 19 sectors for electrode 2, with a finger-type geometry. The disk of the TENG consisted of a rotator and a stator constructed using a laser-cutting machine.

#### 2.1.2. Power Transmission

All gears trained in this study were made of blue nylon with the same modules and used four different types of gears (G1, G2, G3, G4, and a rack gear). All gears had a tooth thickness of 10 mm and a step height of 8 mm. The dimensions of the gears (G1 G2, G3, and G4) were 30 teeth with an outer diameter of 32 mm and an inner hole of 14 mm, 60 teeth with an outer diameter of 62 mm and an inner hole of 10 mm, 30 teeth with an outer diameter of 32 mm and an inner hole of 10 mm, and 15 teeth with an outer diameter of 17 mm and an inner hole of 5 mm, respectively. The rack gear had a dimension of 20 × 20 × 200 mm^3^. A schematic illustration of the gear system is shown in Supplementary Figure S4, and the configuration of the gear system is shown in 3D and 2D in Supplementary Figure S5. Due to being made of nylon material, the gears and rack gears were very light. The rack gear was used as a means of power transmission for converting linear movement to rotation. G1 became a gear clutch by adding a one-way bearing inside. This one-way bearing had a dimension of 10 × 12 × 14 mm^3^. This gear selectively accepts the power transmitted by the rack. To facilitate the rotation, a shaft and lock nut were used.

#### 2.1.3. Designed Circuit Board for Arranging Output and Connected Rotational TENG

The outputs of Unit 1 (U1) and Unit 2 (U2) of the TENG were connected using a circuit board that consisted of components such as a full wave rectifier and capacitors with LEDs. The full wave rectifier was used to invert the output voltage from AC to DC. The PCB board is shown in Supplementary Figure S6.

## 3. Results and Discussion

### 3.1. Device Structure

**Figure 1:**
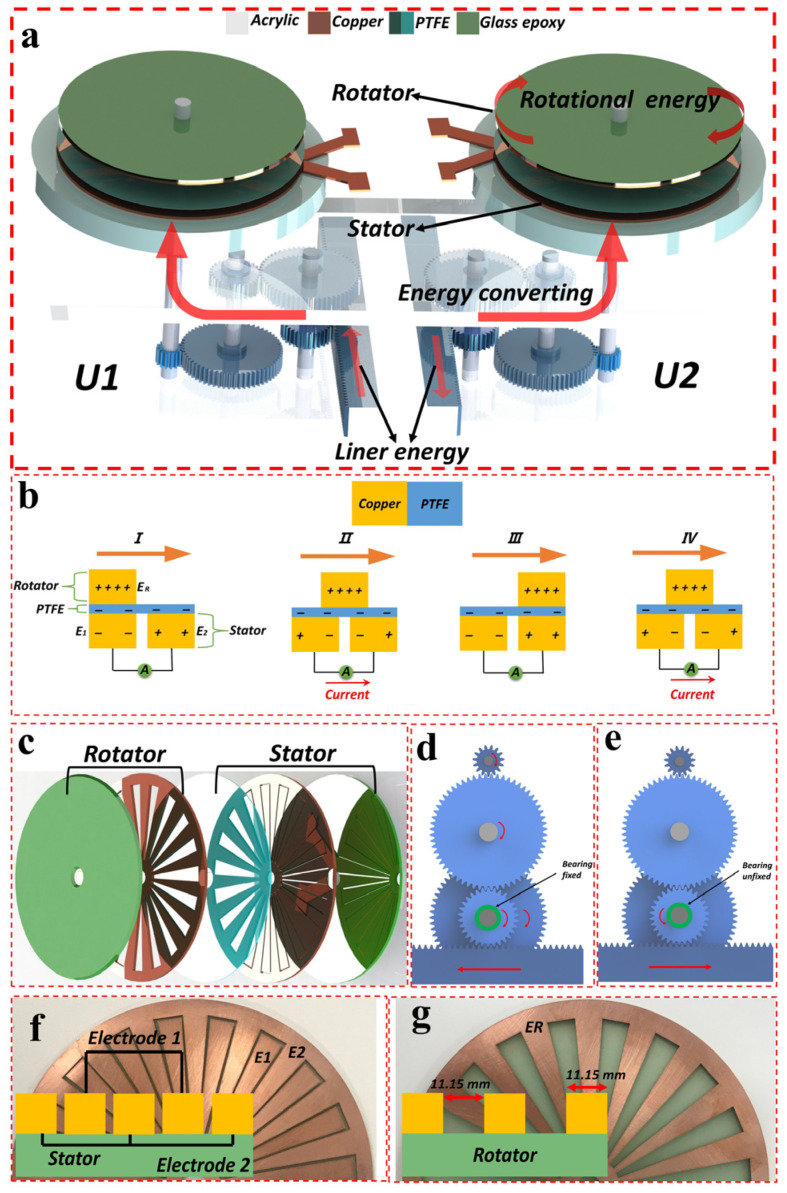
(**a**) Schematic illustration of the DHLR-TENG, which has a force-converting part and an energy-generating part. (**b**) Working principal of the FR-TENG. (**c**) Three-dimensional configuration of the FR-TENG, comprising a stator and a rotator with negative triboelectric materials (PTFE). (**d**,**e**) Schematics of the principle of the one-way bearing, which allows only one-way rotation. (**f**) Surface images of the stator. The 38 Cu sectors. (**g**) Surface images of the rotator. The 19 CU sector units patterned onto the surface of the rotator.

Figure 1a depicts a schematic representation of the design of the DHLR-TENG, which operates via the use of linear mechanical force. The DHLR-TENG consists of two different parts, namely a force-converting part and an energy-generating part, both of which are cube-shaped in structure; Supplementary Figure S12 shows pictorial images of the DHLR-TENG. In the force-conversion process, a button with a column-shaped design is subjected to linear mechanical force. This force is transmitted through a stepper motor and subsequently converted into rotational mechanical force through the use of a gear system. The stepper motor moves forward and backward on Unit 1 and Unit 2, respectively, facilitating this conversion. The transmission of rotating mechanical force to the energy-generating component occurs directly in the alternating states of Unit 1 (U1) and Unit 2 (U2). Upon the removal of the applied linear force on U1, the center of the rack gears reverts back to its initial position via the use of the motor stepper. Figure 1b depicts the structure design and working principal of a freestanding TENG (FR-TENG) with a two-dimensional schematic of the FR-TENG, consisting of a stator and a rotator.

### 3.2. Design and Performance Analysis of DHLR-TENG: Mechanisms for Energy Conversion and Output Optimization

**Figure 2:**
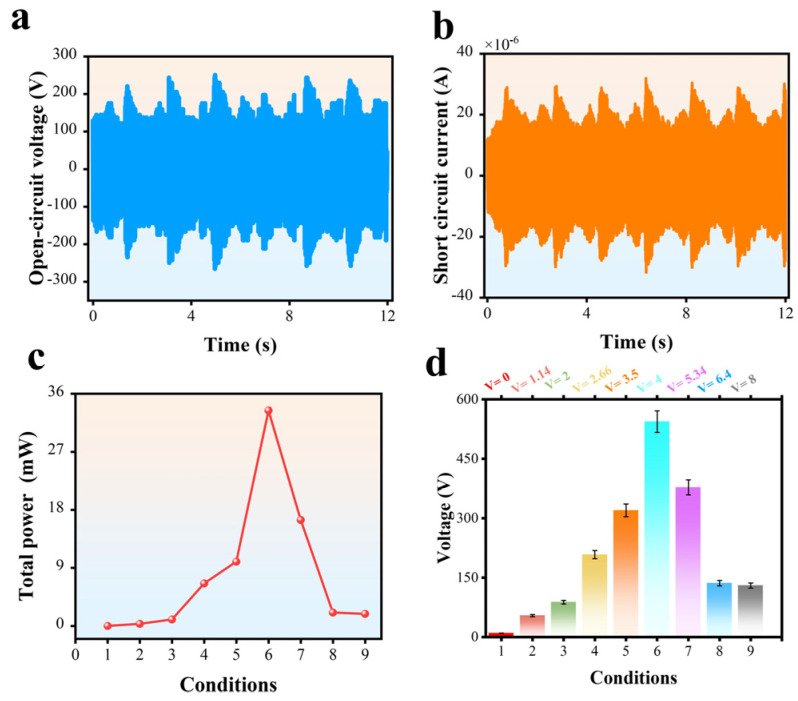
Electrical characterization of the DHLR-TENG, (**a**) Open-circuit voltage (*V_OC_*), (**b**) short-circuit current (I_SC_). (**c**) Schematic illustrating the total power output of the TENG across different operating conditions. (**d**) This bar chart presents the voltage output of the TENG under various conditions at 12 s.

To showcase the considerable output performance, the voltage and current values are typically determined by measuring the total distance travelled during the displacement of the rack gear in 8 cm increments, considered here to be 8 cm forward and backward motion, under the open-circuit and short-circuit conditions, respectively. As depicted in Figure 2a,b, the aforementioned high voltage/current outputs possess the capability to energize red LEDs effectively, hence enabling their application in diverse domains like DHLR-TENG for converting linear mechanical energy into electrical energy effectively to power numerous small electronic devices, such as LEDs. Nevertheless, the actual implementation of TENG for various applications is impeded by the significant impedance mismatch between the TENG and the power management unit, resulting in the generation of only a minimal power output. Therefore, it is imperative to develop a comprehensive and effective optimization approach. The sectors are patterned onto the surface of the stator. Figure 1c depicts the three-dimensional configuration of the FR-TENG, comprising a stator and a rotator with negative triboelectric materials, PTFE. The triboelectric materials consist of the metal electrode E_R_ in the rotator component and the insulator film in the stator. These materials are specifically designed to establish connections with the electrical terminals situated on the side of the stator. The stator was fabricated using a laser-cutting machine developed for the purpose of generating output for the TENG. The E_R_ electrode in the rotator is in a state of electrical buoyancy.

On the other hand, the retrograde linear force resulting from the restorative force of the motor stepper cannot be transmitted to the energy-generating component as a result of the single-directional bearing within the gear system of U1. Conversely, U2 commences the generation of electricity at the terminal point of U1, as depicted in Figure 1d,e. The motor stepper exhibits forward movement for U1 and backward movement. In U1, the linear force imparted to the one-way bearing is converted into a rotating force inside each unit. When the stepper motor is in motion in the forward direction, the one-way bearing located at U1 fails to engage, resulting in the absence of force conversion. The lack of a one-way bearing prevents the generation of a consistent electrical output as well as inhibits the rack gear’s ability to return to its initial position and accept future linear forces in both the forward and backward directions. Supplementary Figure S4 displays the comprehensive specifications of the gear system. The calculation of the number of revolutions of a disk is obtained by multiplying the number of rotations of gear 1 by the rotation ratio of the gear system. Gear 1, having a diameter of 32 mm, undergoes an estimated rotation of 0.867 revolutions when the motor stepper is linearly displaced by a force of 8 cm. The rotational ratio of the whole gear system is roughly 6.92. This means that when gear 1 completes one full revolution, the rotator of the DHLR-TENG completes approximately 6.92 rotations. As an example, upon the application of a linear force, measuring 1 cm, to the button, the rotator disk undergoes an estimated revolution of 0.865 turns. In this study, all measurements were conducted by applying a linear force of 8 cm to depress the button, resulting in an estimated rotation of 6.92 turns for the rotator in the gear configuration. Energy transfer optimization, and calculations of the speeds and ratios are discussed in Supplementary Note S4 and presented in Supplementary Table S1. Within the energy-generating component, a single disk, referred to as the rotator, undergoes rotation due to the transmission of rotational mechanical force from the energy-converting component. Simultaneously, the other disk, known as the stator, remains stationary as it is affixed to the cube-shaped shell. The Supplementary Materials include Video S1, which provides real-time visual representations of the DHLR-TENG procedure. Figure 1f,g depict the surfaces of both the rotator and the stator. The substrate used in this study was composed of glass epoxy material, with a diameter of 150 mm. The surface of the substrate exhibited protruding patterns, which comprised copper (Cu) material. A total of 38 sector units made of copper were arranged in a radial pattern on the stator’s surface, while 19 of these sectors were arranged on the rotator’s surface. The center angle of every sector unit was measured to be 9.47 degrees, while the outermost length was found to be 55 mm in the magnified surface pictures shown in Figure 1f,g, respectively. The separation lengths of the rotator and stator were 11.15 mm and 1 mm, respectively. The Cu pattern on the stator was segmented into two distinct electrodes, namely electrode 1 and electrode 2. The stator was partitioned into 19 sectors for electrode 1 and 19 sectors for electrode 2, with a finger-type configuration. The disk of the TENG was composed of a rotator and a stator, which were fabricated using a laser-cutting machine. Figure 1 illustrates the whole operational mechanism of the DHLR-TENG used for energy generation. In this study, a polytetrafluoroethylene (PTFE) film was affixed to the stator as a triboelectric layer. The purpose of this layer was to facilitate the generation of electrical energy by rotating and sliding with the projecting Cu on the rotator. Following a pre-charging procedure, the PTFE layer on the stator acquires a negative charge, while the Cu surface on the rotator acquires a positive charge, as determined by the triboelectric series. The consistent rotation of the rotator generates an oscillating flow of electric current, known as alternating current (AC), between electrode 1 and electrode 2 for each unit. In addition, as stated before, U1 and U2 were constructed in a similar manner, with identical materials, sizes, and structures. In Figure 2a,b, the relationship V = IR (Ohm’s Law) explains the connection between voltage (V), current (I), and resistance (R) in the Cu-PTFE system and is critical to understanding the observed time-dependent behavior. The voltage generated across the interface is directly proportional to the current flowing through the circuit, assuming a constant resistance. However, during sliding, the voltage and current are not static and varies over time due to the initial and final velocities of each disk. This variation led to fluctuations in both voltage and current. The effect of load resistance on the voltage and current output under consistent mechanical energy in this experiment has been investigated in Supplementary Note S1 and Supplementary Figures S8 and S9.

### 3.3. Electricity Generation Process

The triboelectric materials in this system consist of a metal electrode (E_R_) in the rotator and an insulating layer attached to the surface of the stator, which contains two separate electrodes (E_1_ and E_2_). Both the rotator and stator components were fabricated using laser-cutting technology to ensure precise matching and alignment. These electrodes are connected to the electrical terminals, facilitating the TENG output, as shown in Figure 1b. Initially, in Step I, the E_R_ electrode overlaps with E_1_, causing the triboelectric materials to acquire equal but opposite charges due to electrostatic forces in the freestanding structure. As the rotator begins turning rightward in Step II, E_R_ starts to partially overlap with both E_1_ and E_2_, initiating triboelectrification through direct contact. This results in negative charges accumulating on the PTFE surface and positive charges on the metal, with the positive charge density on the rotator being twice that of the stator due to unequal contact areas. The rightward rotation continues in Step III, causing further movement of the E_R_ electrode between E_1_ and E_2_, leading to an electron flow from E_1_ to E_2_ through the external circuit, driven by electrostatic imbalance. Finally, in Step IV, the E_R_ electrode overlaps once again with E_1_, causing the electron flow to reverse from E_2_ back to E_1_. This cyclical motion generates an oscillating electrical current or voltage signal, which fluctuates in sync with the rotational movement, resulting in a continuous output of electrical energy from the triboelectric nanogenerator (FR-TENG).

In the open-circuit condition, electrons cannot flow between the electrodes. The open-circuit voltage ($V_{OC}$) is defined as the electric potential difference between the two electrodes, expressed as $V_{OC}$ = $V_{E1}$ − $V_{E2}$. In Step I, E_1_ reaches its maximum potential while E_2_ reaches its minimum potential, resulting in the maximum $V_{OC}$. As the rotator begins to spin, this voltage gradually decreases. Once the rotator passes the midpoint, $V_{OC}$ with an opposite polarity starts to form and continues to build until the rotator reaches Step III. Beyond this point, the $V_{OC}$ changes direction due to the periodic structure. Based on the assumption that the dielectric layer’s thickness is much smaller than the width shown in Figure 2b, an analytical model can be created. In this model, any overlap between the rotator and the electrodes is treated as a parallel plate capacitor, disregarding edge effects. Using Gauss’s Theorem, the $V_{OC}$ for each unit (*U*1 and *U*2) can be analytically calculated, with detailed information provided in Supplementary Figure S10 and Supplementary Note S2.

(1) $$Step\;I:\;{V_{OC}}_{\left(initial\right)}=V_{E1}{-V}_{E2}=\frac{2d\cdot \sigma }{\varepsilon_{0}\varepsilon_{r}}$$

(2) $$Step\;II:{\;V}_{OC}\left(\theta \right)=V_{E1}{-V}_{E2}=\frac{d\cdot \sigma }{\varepsilon_{0}\varepsilon_{r}}\left(\frac{\theta }{\theta_{0}-\theta }-\frac{\theta_{0}-\theta }{\theta }\right)\;$$

(θ approaches neither 0 nor $\theta_{0}$)

(3) $$Step\;III:\;{V_{OC}}_{\left(final\right)}=V_{E1}{-V}_{E2}=-\frac{2d\cdot \sigma }{\varepsilon_{0}\varepsilon_{r}}$$

where d is the thickness of the PTFE layer, σ is the triboelectric charge density on top of the PTFE layer, $\varepsilon_{0}$ is the dielectric constant of vaccum, $\varepsilon_{r}$ is the relative dielectric constant of PTFE, α is the angle at which the rotator rotates away from the initial state, and $\alpha_{0}$ is the central angle of a single rotator unit. Equation (2) can only be used to illustrate the changing trend of the $V_{OC}$ (see Supplementary Note S2).

The theoretical peak-to-peak value of the $V_{OC\;p-p}$ for each *U* needs to be calculated by subtracting Equation (3) from Equation (1):

(4) $$V_{OC,p-p}=V_{E1}{-V}_{E2}=\frac{4d\cdot \sigma }{\varepsilon_{0}\varepsilon_{r}}$$

Additionally, to calculate the open-circuit voltage peak to peak in this system (${Vs}_{oc\;p-p}$), substitute Equations (S24) and (S25) into Equation (S23) (see Supplementary Note S2):

(5) $${Vs}_{oc\;p-p}(t)=\left\{\begin{array}{c} V_{U1}(t),\;0\;\leq t_{p}<\frac{t_{p}}{2}\\ V_{U2}(t),\;\frac{t_{p}}{2}\;\leq t_{p}<t_{p} \end{array}\right.$$

where $V_{U1}(t)$ and $V_{U2}(t)$ output the voltage peak to peak for Unit 1 and Unit 2, respectively, and $t_{p}$ is the time period modulated by 4 s (so the behavior repeats every 4 s). This diagram displays the voltage required to select *V_(p−p)_* for *U*1 and *U*2, as well as the impact of different time intervals on *U*1 and *U*2. The maximum voltage of $V_{U1}$ is determined to be 160 V, while the minimum voltage is observed at −152 V, resulting in a $V_{U1,}$_(p−p)_ = 312 V. Conversely, the upper limit of voltage, denoted as $V_{U2}$, is established at 160 V, while the lower limit is seen at −168 V, resulting in a value of $V_{U1,}$_(p−p)_ = 328 V. $V_{U1}$ and $V_{U2}$ are shown in Supplementary Figure S1. The combined maximum voltage, taking into consideration $V_{U1}$ and $V_{U2}$, is 280 V, while the minimum voltage is −264 V. This results in a peak-to-peak voltage value of 544 V for the entire system. According to Figure 2, the energy generation time for each $V_{U}$ is 2 s. The outcomes of this procedure necessitate a duration of 4 s for both cycles, namely $V_{U1}$ and $V_{U2}$. When this process persists and repeats, it leads to the generation of a complete cycle of alternate current when the outputs of $V_{U1}$ and $V_{U2}$ are coupled. Furthermore, the voltage generation process occurs within a single vibration of the motor stepper, which corresponds to a single frequency of movement.

The open-circuit voltage peak to peak (*Vs_OC,p−p_*) and the short-circuit current peak to peak (I_SC,p−p_) of the DHLR-TENG were measured under the nine conditions at a vibration frequency of 3 Hz. The voltage and current signal for 1 Hz are shown in Supplementary Figure S3. The maximum electrical outputs of the DHLR-TENG, with a *V_OC_* value of 544 V, an I_SC_ value of 61.16 μA, and a power of 33.27 μW, were achieved under Condition 6, as shown in Figure 2a,b and c, respectively.

The bar chart in Figure 2d illustrates the relationship between nine distinct operating conditions and their corresponding voltage outputs, with each condition characterized by a specific velocity value. These results emphasize the versatility of the TENG in energy harvesting, demonstrating its capability to function effectively across a broad range of mechanical inputs, from low-velocity vibrations to high-speed motions. This adaptability makes the TENG a viable solution for capturing and converting energy from diverse environmental sources, ensuring consistent energy output even under fluctuating conditions. The conditions, labeled 1 through 9, represent velocities ranging from 0 cm/s to 8 cm/s. Specifically, Condition 1 exhibits the lowest velocity of 0 cm/s, resulting in a voltage of 0 V, while Condition 2 presents a velocity of 1.14 cm/s and a corresponding voltage of 54 V. Condition 3 follows with a velocity of 2 cm/s and a voltage of 88 V. Condition 4 shows a velocity of 2.66 cm/s, producing a voltage of 208 V. Condition 5, with a velocity of 3.5 cm/s, generates a voltage of 320 V. Condition 6, operating at a medium velocity of 4 cm/s, yields the highest voltage of 544 V. Condition 7 presents a velocity of 5.34 cm/s, with an associated voltage of 378 V. Condition 8 shows a velocity of 6.4 cm/s and a voltage of 136 V, while Condition 9, with the highest velocity of 8 cm/s, produces a voltage of 130 V.

Upon observing the bar chart, a discernible trend emerges between velocity and voltage. Initially, from Condition 1 to Condition 5, there is a notable rise in both velocity and voltage, suggesting a positive correlation between the two variables. This trend continues with Condition 6, where both velocity and voltage exhibit further increments. However, from Conditions 7 to 9, despite an increase in velocity, the voltage value experiences a slight decrease compared to Condition 6. Finally, in Condition 9, although the velocity reaches its peak at 8 cm/s, there is a noticeable decrease in voltage compared to the preceding condition. Overall, the bar chart provides a visual representation of the relationship between velocity and voltage across different conditions, starting from 0 cm/s (stationary) to 8 cm/s. While there appears to be a general trend of increasing voltage with higher velocities, the relationship is not strictly linear. The output voltage increases until the velocity reaches 4 cm/s, showing a correlation with rotator speed. However, from Conditions 7 to 9, despite an increase in velocity, the voltage decreases.

**Figure 3:**
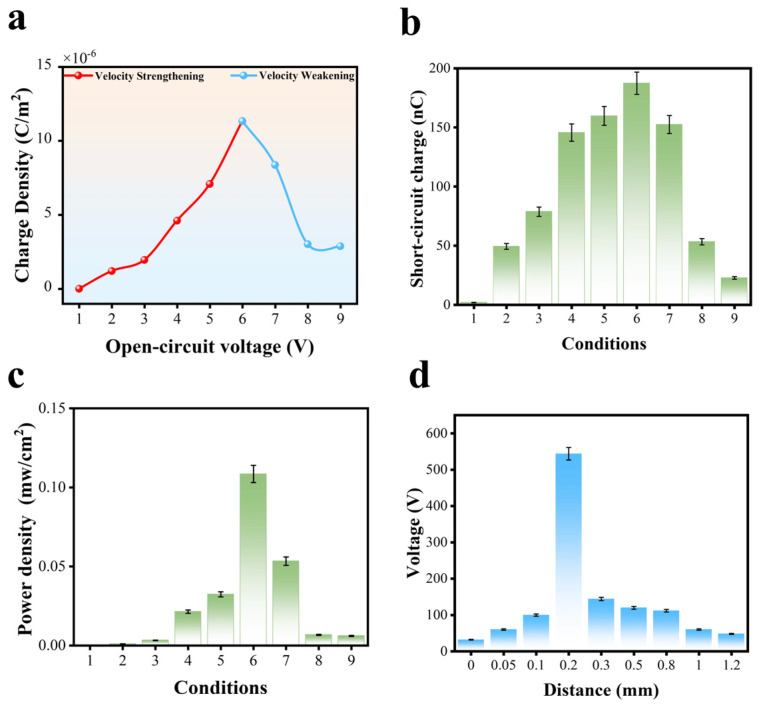
(**a**) The relationship between charge density and nine different conditions under velocity strengthening and velocity weakening. (**b**) Short-circuit charge measured under nine different conditions. (**c**) Power density output per square centimeter (mW/cm^2^) generated under nine different conditions. (**d**) Open-circuit voltage generated at different distances between the rotator and the PTFE surface (TENG).

Equation (5), used to estimate the surface charge density of the PTFE film in the DHLR-TENG, calculates a charge density of 11.33 µC/m^2^ at a ${Vs}_{OC,p-p}$ value of 544 V, as shown in Figure 3a. The interaction between Cu-PTFE during sliding, illustrated in Figure 3a, exhibits distinct frictional behaviors: velocity strengthening and velocity weakening. Velocity strengthening, represented by the red line, occurs when the frictional resistance between Cu-PTFE increases with sliding velocity. This results in a stable rise in charge density, peaking at 11.33 µC/m^2^ under Condition 6, before gradually declining as the system stabilizes. In contrast, velocity weakening, depicted by the blue line, is characterized by a reduction in frictional resistance as the sliding velocity increases. This behavior is reflected in an initial rise in charge density that peaks earlier, at 7.08 µC/m^2^ under Condition 5, followed by a rapid decline. These contrasting behaviors underscore the dynamic frictional properties of the Cu-PTFE interface, where velocity strengthening fosters stability, while velocity weakening leads to instability at higher sliding velocities. This graph highlights the relationship between surface charge density and open-circuit voltage across different velocities, indicating that higher velocities generally result in higher charge densities. Notably, the charge density increases with sliding velocity, reaching a maximum at 4 cm/s, a phenomenon attributable to velocity strengthening. These results demonstrate that while the velocity increases linearly, the output voltage does not follow a similar trend. This nonlinearity can be attributed to the mechanical effects associated with higher rotational speeds in the TENG sliding system.

At increased speeds, the centrifugal forces and vertical vibrations of the rotor become more pronounced. These effects are exacerbated by imperfections in the fabrication process, which lead to an increase in the gap between the rotor and stator. As a result, the maximum output charge generated by the system declines with increasing rotational speed. This phenomenon explains the observed decrease in output voltage, despite the linear increase in velocity. The findings emphasize the importance of precision in the fabrication process to minimize variations in the gap between the rotor and stator, particularly at higher rotational speeds. Addressing these mechanical limitations could enhance the overall efficiency of the TENG system for high-speed applications.

Figure 3b shows the short-circuit charge (Q_SC_) of the DHLR-TENG transferred by one cycle of vibration. To compare an I_SC_ value directly with the corresponding Q_SC_, the Q_SC_ value was calculated using the time interval integral of I_SC_. The trend of Q_SC_ according to each condition is identical to those of *V_OC_* and I_SC_. In Condition 6, the maximum value of Q_SC_ was approximately 187.4 nC for one cycle of vibration. Furthermore, power density is calculated by dividing the maximum power output by the area over which the power is distributed, as shown in Figure 3c. The highest value, determined under Condition 6, is 0.11 mW/cm^2^. On the other hand, according to the geometries of the DHLR-TENG, the volume of the DHLR-TENG is determined to be **193.865** cm^3^. Hence, the power density per volume is **171.616** W/(m^3^·Hz). These results highlight the DHLR-TENG’s exceptional performance among energy-harvesting TENGs. A detailed comparison is provided in Supplementary Table S2.

Moreover, Figure 3d illustrates the relationship between *V_OC_* and the distance (x) between the rotator and the PTFE surface, revealing an inverse correlation between these variables. The separation distance (x) can be adjusted to regulate the horizontal force necessary for movement, as illustrated in Figure 1a. Distance (x) refers to the gap between the rotator and the surface of the stator. Strong sliding is facilitated by a high frictional force with a small x, which is the result of high pressure. A low frictional force is generated by low pressure, which triggers feeble sliding due to a large x. The nine conditions of x were used to measure the electrical outputs of the DHLR-TENG. The smallest x was found in Condition 1, while the greatest x was found in Condition 9. x increases linearly from Condition 1 to 9. The value of x is approximately 0 mm to 1.2 mm when Conditions 1 to 9 are met. As a result, the sliding of the two disks becomes more robust as the value of x decreases; however, the rack gear requires a greater amount of mechanical force to be driven. It is anticipated that an optimal condition for x will result in the highest possible power output. The DHLR-TENG’s *V_OC_* was measured at a vibration frequency of 3 Hz under the nine conditions. In Condition 4, the DHLR-TENG attained its highest electrical output, with a *V_OC_* value of 544 V, as shown in Figure 3d. A large x value resulted in ineffectual gliding beyond Condition 4. The DHLR-TENG’s electrical output was reduced as a result of a low x value in Condition 4.

### 3.4. Durability

**Figure 4:**
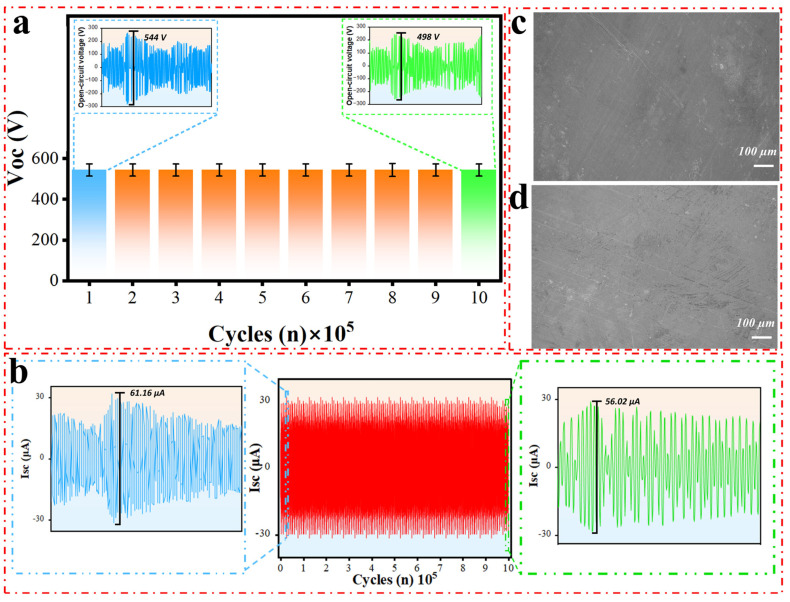
Stability and durability performance: (**a**,**b**) open-circuit voltage and short-circuit current in the DHLR-TENG over 1,000,000 cycles. (**c**,**d**) Comparison of the scanning electron microscope (SEM) images of the PTFE before and after 1,000,000 cycles.

The durability of the TENG in this study has been thoroughly tested, showcasing exceptional stability and efficiency even after 1,000,000 cycles of continuous operation. A key metric for evaluating the stability of TENGs is their ability to sustain a stable electrical output over extended periods. As shown in Figure 4a,b, the voltage and current waveforms after 1,000,000 cycles exhibit only minor changes, with the voltage decreasing from 544 V to approximately 498 V and the current stabilizing at around 56.02 µA. This slight reduction, representing about a 9% change in output, highlights the robustness of the system and its ability to efficiently convert mechanical energy into electrical energy over extended periods with minimal degradation. A scanning electron microscope (SEM) analysis of the PTFE surface before and after 1,000,000 cycles, as shown in Figure 4c,d, reveals noticeable changes in surface morphology. These changes suggest that mechanical wear, while minimal, can affect the output capability of the triboelectric layer over time. Although the degradation of output performance caused by device abrasion remains a challenge, the structural design implemented in this study has significantly improved the output stability of the DHLR-TENG. The DHLR-TENG system demonstrates excellent long-term reliability, with its output remaining relatively stable despite minor decreases, making it a promising solution for sustained energy-harvesting applications.

Environmental factors, including temperature and humidity, are known to significantly influence the performance of TENGs. Elevated temperatures can amplify the electron thermionic emission effect, resulting in charge loss, while high humidity introduces water molecules that interact with the electrode surface, reducing surface charge density and output performance [24,25]. Such factors, along with other environmental conditions, play a critical role in determining the stability, efficiency, and long-term reliability of TENG systems.

The consistent performance observed, with minimal degradation even after 1,000,000 cycles, is a notable achievement that reinforces the TENG’s potential for robust and long-lasting energy-harvesting solutions. Combined with insights from prior studies on environmental influences and the SEM analysis of surface wear, the results highlight the TENG’s suitability for long-term use in various energy-harvesting applications, making it a viable and sustainable solution for powering small electronic devices and sensors.

### 3.5. Application

**Figure 5:**
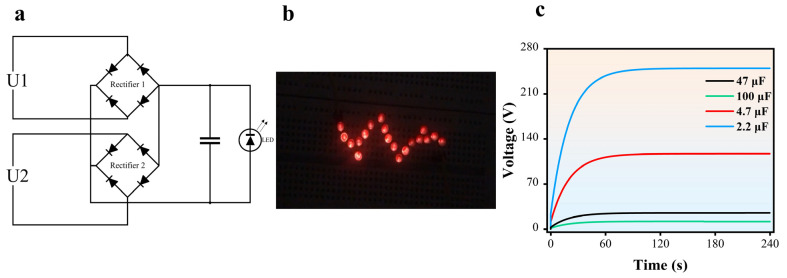
(**a**) Circuit diagram including the DHLR-TENG, a capacitor, and commercial LEDs. (**b**) Optical photograph of red commercial LEDs showing that the red commercial LEDs were lit up simultaneously and continuously when powered by the DHLR-TENG. (**c**) Voltage curves of capacitor charging at 4 cm/s for 240 s.

The free-standing triboelectric nanogenerator (FR-TENG) has a notable practical use in providing power to small electronic devices such as light-emitting diodes (LEDs) through a continuous direct current (DC) output. The operational principle of the TENG is centered on the transformation of mechanical energy into electrical energy by the initiation of frictional interaction between two separate materials. The TENG effectively harnesses and converts mechanical energy, resulting in a uniform output voltage in the form of direct current. This direct current is capable of providing uninterrupted illumination to an LED, ensuring a consistent and uninterrupted light source. The presented practical demonstration of the TENG technology showcases its considerable potential as an autonomous energy source, specifically well suited for sustaining low-power electronic devices. TENGs provide a sustainable and eco-friendly option for powering devices in various environments. They offer a forward-thinking solution for energy generation and usage, enabling more efficient and environmentally sensitive electronic applications. The output voltage possesses the capability to illuminate some LEDs consistently and continuously, as shown in the Supplementary Materials, including Video S2, and Supplementary Figure S7 and Figure 5b. This is attributed to the alternative current generated by this study, which is subsequently utilized to illuminate some LEDs.

In order to demonstrate the capabilities of the DHLR-TENG as an energy provider, a capacitor and commercially available LEDs were linked to the DHLR-TENG using a full wave rectifier, as seen in Figure 5a. In addition, the DHLR-TENG powered the red commercial LEDs, causing them to illuminate simultaneously, as seen in Figure 5b. The DHLR-TENG is very efficient at converting mechanical energy into electrical energy. The capacitor linked to the DHLR-TENG saw rapid charging when the DHLR-TENG was activated, as seen in Figure 5c. The voltage across the capacitors, with a capacitance of (100, 47, 4.7, 2) µF, on the 4.7 µF surpassed 110 V after 240 s. The DHLR-TENG has been shown to efficiently transfer linear mechanical energy into electric energy, making it suitable for powering tiny electronic devices.

## 4. Conclusions

In conclusion, this study has demonstrated a logical approach to enhancing the longevity of TENG by combining a radially arranged TENG with a transmission mechanism, enabling uninterrupted functioning for extended periods of time. This research demonstrated the disk-based DHLR-TENG, which utilizes a gear system to transform linear mechanical energy into rotational energy. To generate a complete cycle of alternating current, two units of TENG were used. These units can be connected to an electric board to combine their outputs and convert AC into DC signals. When an equal velocity is applied in both the forward and backward directions for each unit, a complete cycle of alternating current is generated with an open-circuit voltage (*V_OC_*) value of 544 V, a short circuit current (I_sc_) value of 61.16 µA, a maximum power of 33.27 mW, and a durability of more than 1,000,000 cycles. Due to its exceptional electrical properties, the DHLR-TENG can efficiently provide a substantial quantity of electrical energy to a wide range of tiny electronic devices such as LEDs.

### Electrical Measurements

The Haijie Jiachuang LH4572 stepper motor was used for linear movement between Unit 1 and Unit 2. This motor is equipped with a synchronous belt, a sliding module, high-speed accuracy, quiet operation, and dustproof capabilities. It has a 57-stepper and a stroke length of 200 mm. A function generator was used to produce an electrical signal with a precisely regulated amplitude. An electrodynamic shaker was then used to translate the electrical signals into consistent linear mechanical motion. Subsequently, the DHLR-TENG used this linear mechanical motion for both forward and backward movement. An oscilloscope **(Tektronix (MDO3032), Beaverton, OR, USA)** used to test the electrical signal of the TENG, including the output voltage and short-circuit current. The oscilloscope had an inbuilt load resistance of 100 MΩ. Then, a Pico ammeter **(Keysight (B2981A), Santa Rosa, USA)** was used to measure the short-circuit current, and a scanning electron microscope **(ZEISS (GeminiSEM300), Oberkochen, Germany)** was used to scan the surface of the PTFE.

## Data Availability

The data that support the findings of this study are available from the corresponding author upon reasonable request.

## Supplementary Materials

The following supporting information can be downloaded at: https://www.mdpi.com/article/10.3390/mi16010011/s1, Figure S1: The provided diagram illustrates the correlation between Open-circuit voltage and Time for both unit 1 and unit 2 at 12 s; Figure S2: (a) Surface images of the stator. Cu of 38 sectors is patterned onto the surface of the stator. (b) Surface images of the rotator. Cu of 19 sectors is patterned onto the surface of the rotator; Figure S3: (a) the voltage signal and (b) current signal in 1Hz; Figure S4: Schematic illustration of the gear system and detailed parameters such as a diameter and number of teeth; Figure S5: (a) the three-dimensional graphic of the gears system. (b) the two-dimensional graphic of pairs gears; Figure S6: A printed circuit board (PCB) which use for output combination and converting alternative current to direct current; Figure S7: The connecting circuit and Optical photograph of the red commercial LEDs was lit up; Figure S8: Generated electrical energy of the DHLR-TENG: resistance dependency of the output voltage and the output current of DHLR-TENG; Figure S9: Resistance dependency of the output power of the DHLR-TENG; Figure S10: Schematic illustration of a cross-sectional view of charge distribution in open-circuit condition at the intermediate state; Figure S11: Schematic illustration of a cross-sectional view of charge distribution in open-circuit condition at the initial state; Figure S12: The pictorial images of (a) top, (b) right, (c) back, and (d) front side of the device; Table S1: Show the details of the gears speed; Table S2: The comparison of power density and volume of previously reported TENGs for energy harvesting; Video S1: The DHLR-TENG operated by the motor stepper; Video S2: The red commercial LEDs lit up continuously by DHLR-TENG; Note S1: The effect of load resistance on the voltage and current output of the DHLR-TENG; Note S2: Theoretical analysis of operating process in open-circuit condition; Note S3: Theoretical analysis of operating process in short-current condition; Note S4: Gear configuration and energy transfer, optimization of energy transfer and calculations of speeds and ratios. References [26,27,28,29,30,31,32,33,34,35,36,37,38,39,40] are cited in Supplementary material.
