# Supplementary material for: Generating a Full Cycle of Alternative Current Using a Triboelectric Nanogenerator for Energy Harvesting
Source: Micromachines (Basel). 2024 Dec 25;16(1):11. doi: 10.3390/mi16010011 (PMC11767832; doi:10.3390/mi16010011)
Supplement: Supplementary file 1 [file micromachines-16-00011-s001.zip › micromachines-3317068.pdf]

Supporting Information

Generating Full Cycle of Alternative Current by using Triboelectric Nanogenerator for Energy Harvesting

Aso Ali Abdalmohammed Shateri[1,2], Fengling Zhuo[1], Nazifi Sani Shuaibu[1], Rui Wan[1], Liangquan Xu[1], Dinku Hazarika[1], Bikash Gyawali[3], , Xiaozhi Wang[1]*

1. Zhejiang University College of Information Science and Electronic Engineering, Hangzhou 310027, Zhejiang Province, China.
2. University of Garmian College of Education, Physics Department, Kalar 46021, Kurdistan Region, Iraq.
3. Zhejiang University College of electrical engineering, Hangzhou 310058, Zhejiang Province, China.

*Corresponding authors: Xiaozhi Wang; xw224@zju.edu.cn

Table of contents

Supplementary Figure S1 | The provided diagram illustrates the correlation between Open-circuit voltage and Time for both unit 1 and unit 2 at 12 second. 4

Supplementary Figure S2 | (a) Surface images of the stator. Cu of 38 sectors is patterned onto the surface of the stator. (b) Surface images of the rotator. Cu of 19 sectors is patterned onto the surface of the rotator...4

Supplementary Figure S3 | (a) the voltage signal and (b) current signal in 1Hz 5

Supplementary Figure S4 | Schematic illustration of the gear system and detailed parameters such as a diameter and number of teeth. 5

Supplementary Figure S5 | (a) the three-dimensional graphic of the gears system. (b) the two-dimensional graphic of pairs gears. 6

Supplementary Figure S6 | A printed circuit board (PCB) which use for output combination and converting alternative current to direct current. 6

Supplementary Figure S7 | The connecting circuit and Optical photograph of the red commercial LEDs was lit up..7

Supplementary Figure S8 | Generated electrical energy of the DHLR-TENG: resistance dependency of the output voltage and the output current of DHLR-TENG. 8

Supplementary Figure S9 | Resistance dependency of the output power of the DHLR-TENG. 8

Supplementary Figure S10 | Schematic illustration of a cross-sectional view of charge distribution in open-circuit condition at the intermediate state. 9

Supplementary Figure S11 | Schematic illustration of a cross-sectional view of charge

distribution in open-circuit condition at the initial state. 9

Supplementary Figure S12 | The pictorial images of (a) top, (b) right, (c) back, and (d) front side of the device. 20

Supplementary Note S1 | The effect of load resistance on the voltage and current output of the DHLR-TENG. 7

Supplementary Note S2 | Theoretical analysis of operating process in open-circuit condition 10

Supplementary Note S3 | Theoretical analysis of operating process in short-current condition. 12

Supplementary Note S4 | Gear configuration and energy transfer, optimization of energy transfer and calculations of speeds and ratios. 15

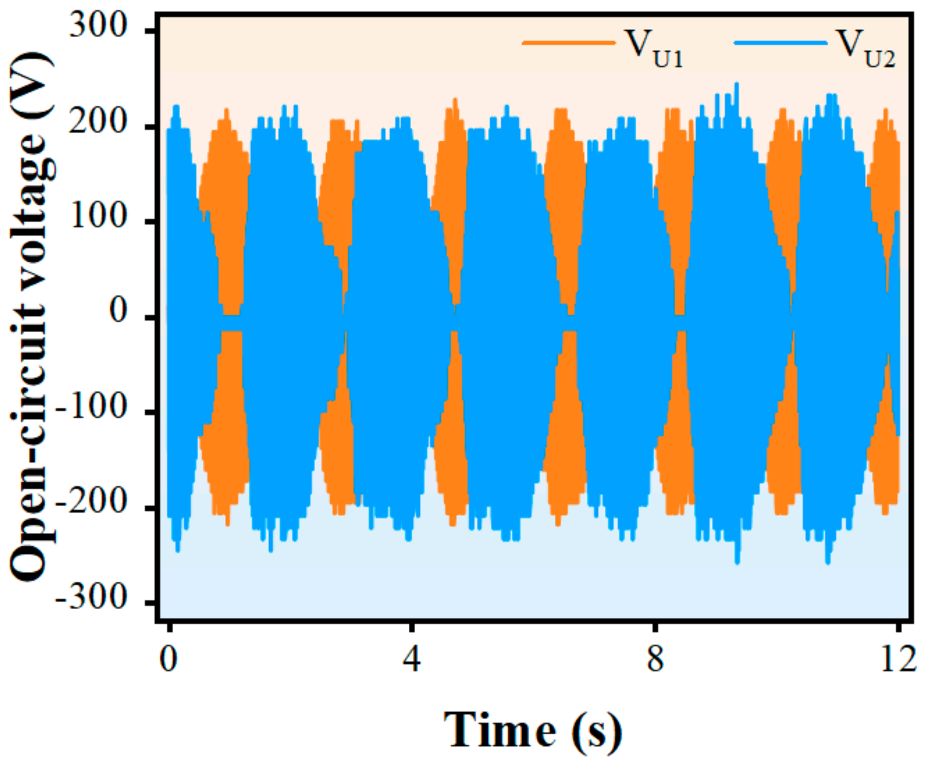

Supplementary Figure S1│ The provided diagram illustrates the correlation between Open-circuit voltage and Time for both unit 1 and unit 2 at 12 second.

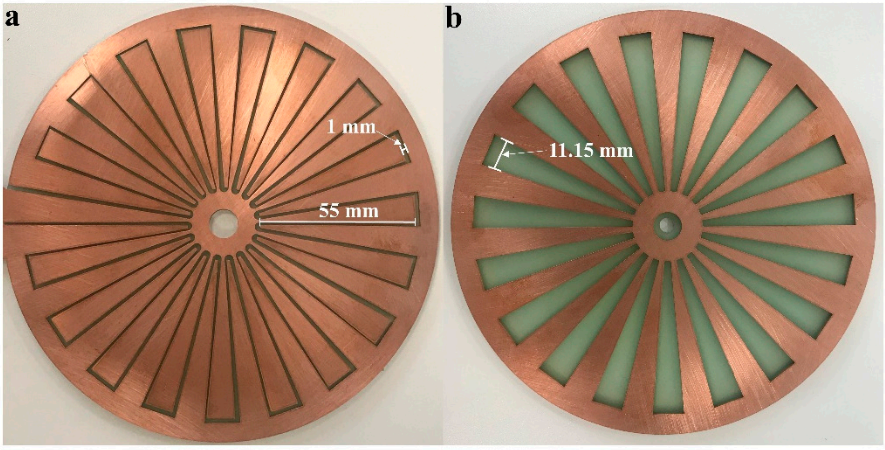

Supplementary Figure S2│(a) Surface images of the stator. Cu of 38 sectors is patterned onto the surface of the stator. (b) Surface images of the rotator. Cu of 19 sectors is patterned onto the surface of the rotator.

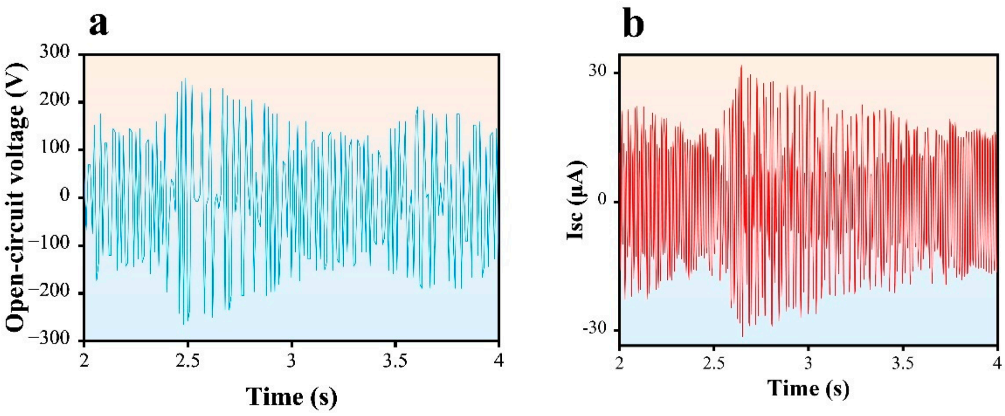

Supplementary Figure S3 | (a) the voltage signal and (b) current signal in 1Hz

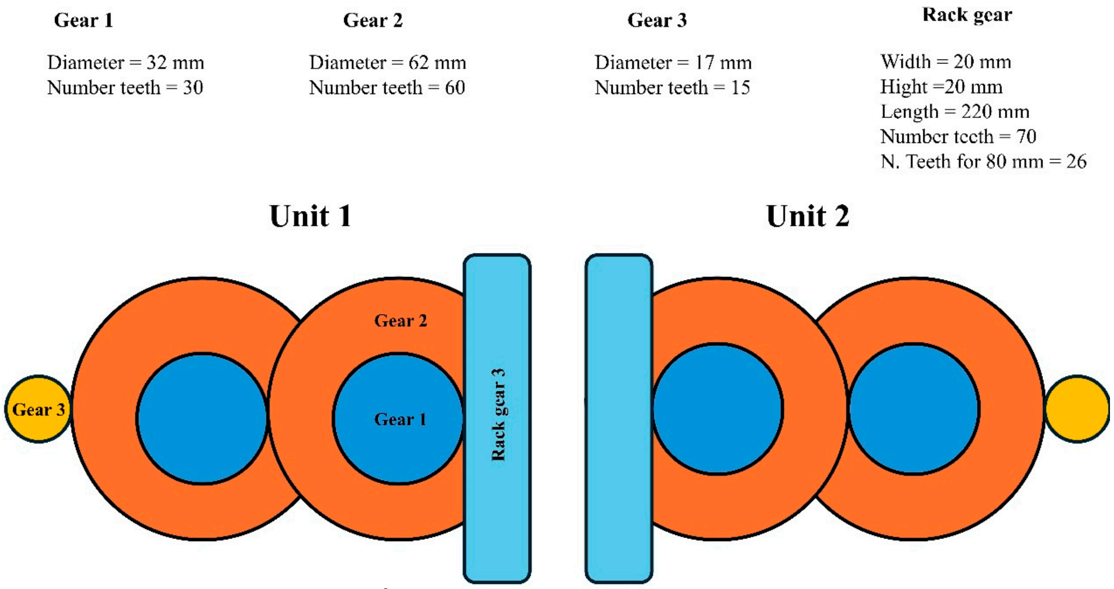

Supplementary Figure S4 | Schematic illustration of the gear system and detailed parameters such as a diameter and number of teeth.

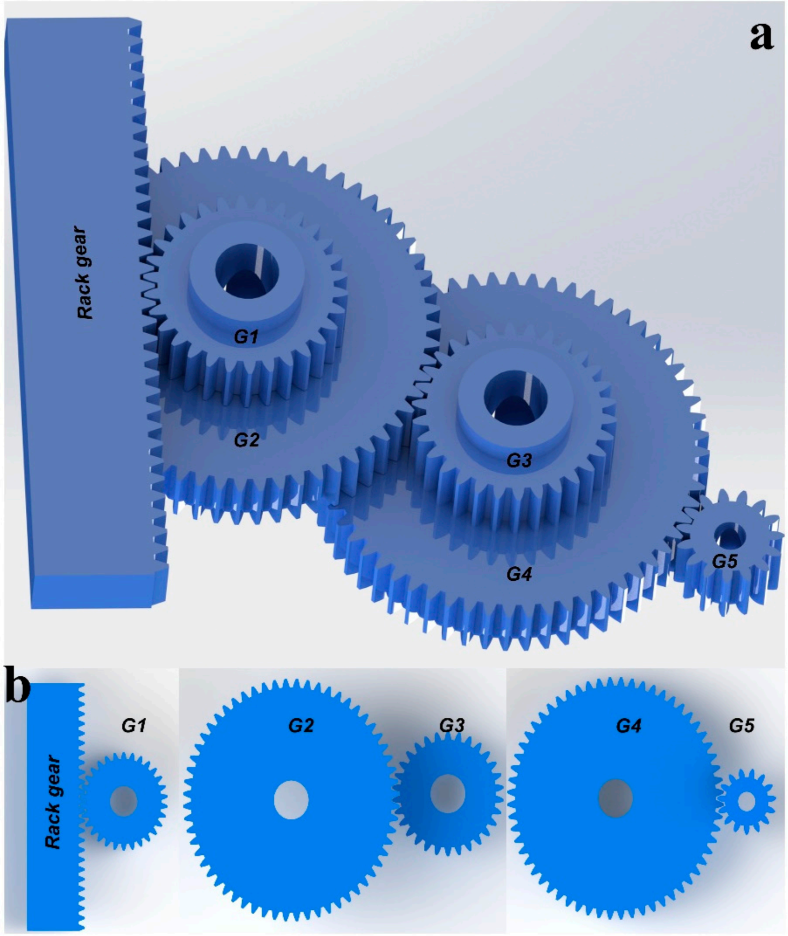

Supplementary Figure S5 | (a) the three-dimensional graphic of the gears system. (b) the two-dimensional graphic of pairs gears.

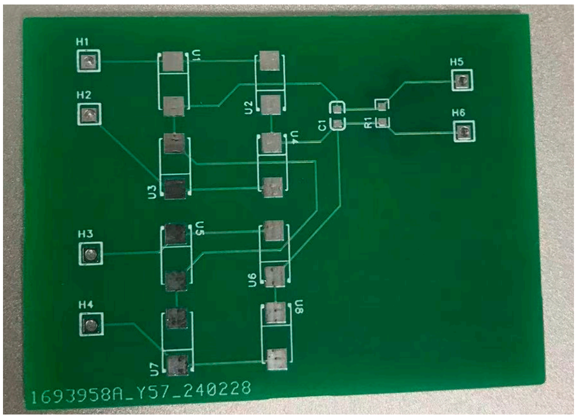

Supplementary Figure S6 | A printed circuit board (PCB) which use for output combination and converting alternative current to direct current.

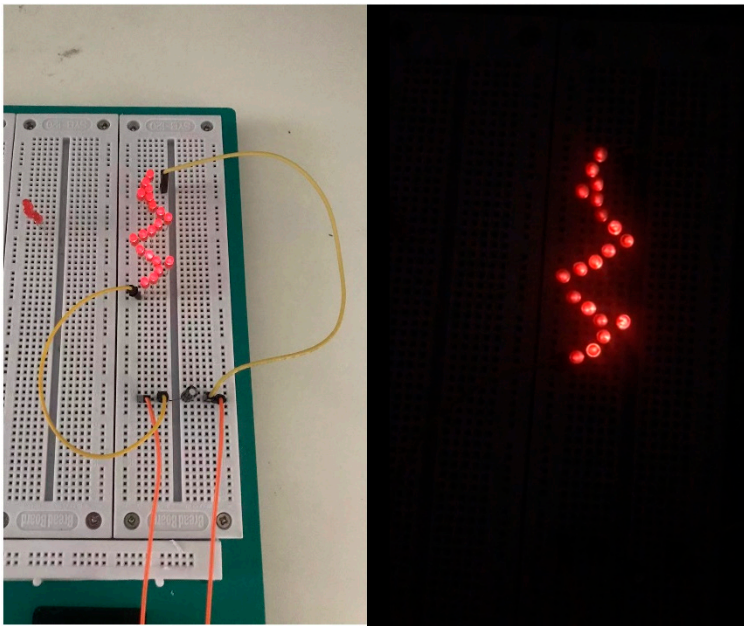

Supplementary Figure S7 | The connecting circuit and Optical photograph of the red commercial LEDs was lit up.

Supplementary Note S1 | The effect of load resistance on the voltage and current output of the DHLR-TENG.

The effect of load resistance on the voltage and current output under consistent mechanical energy in this experiment has been investigated. Up to a load resistance of 500 kΩ, the maximum output current remained steady at 61.16 μA, while the voltage was low due to the device's high internal impedance. Beyond ~9 MΩ, the current decreased, and the voltage increased steadily, following Ohm's Law. This inverse relationship between current and voltage highlights the role of load resistance in determining output characteristics.

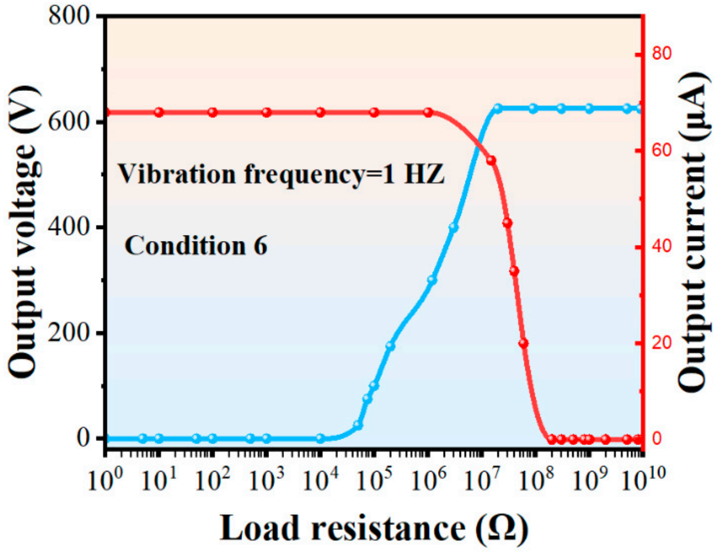

Supplementary Figure S8│Generated electrical energy of the DHLR-TENG: resistance dependency of the output voltage and the output current of DHLR-TENG.

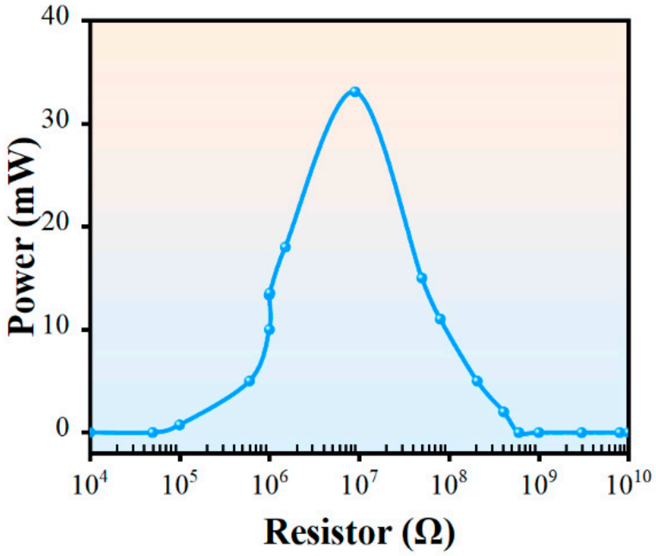

Supplementary Figure S9│Resistance dependency of the output power of the DHLR-TENG.

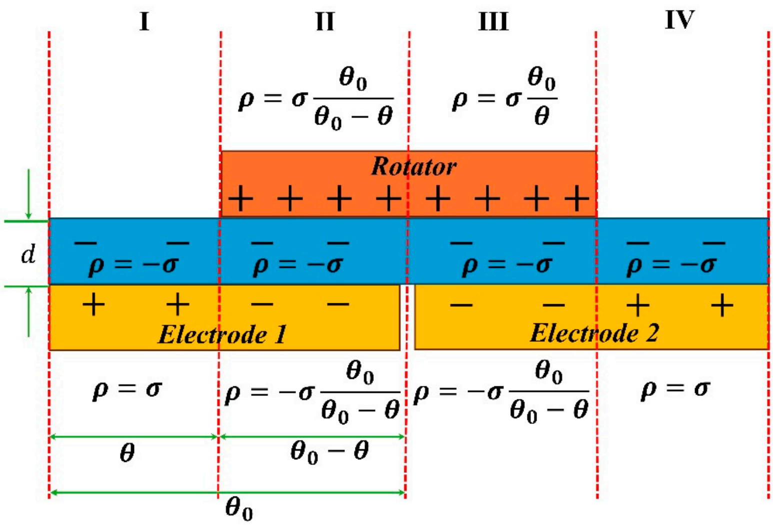

Supplementary Figure S10 | Schematic illustration of a cross-sectional view of charge distribution in open-circuit condition at the intermediate state.

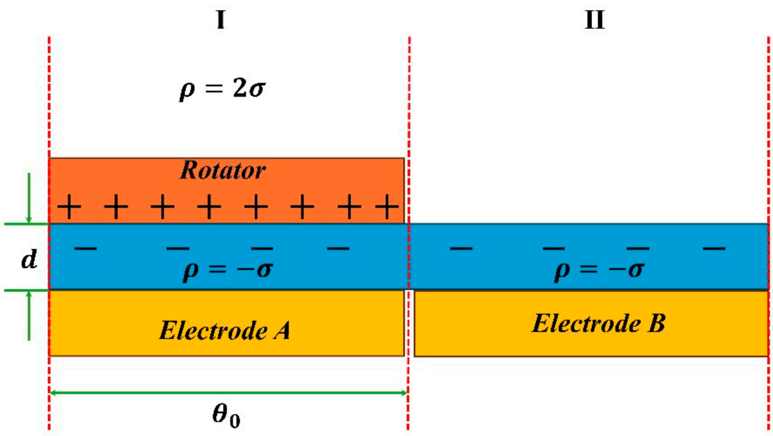

Supplementary Figure S11 | Schematic illustration of a cross-sectional view of charge distribution in open-circuit condition at the initial state.

Supplementary Note S2│Theoretical analysis of operating process in open-circuit condition.

Based on the assumption that the thickness of the dielectric layer (PTFE) is far smaller than its width feature, a simplified model can be used in which any overlapped region between the rotator and the electrodes can be treated as a parallel-plate capacitor without consideration of edge effect. With triboelectric charge density of $-\sigma$ on the PTFE surface, the non-overlapped regions on electrode 1 and electrode 2 (regions 1 and 4 in Supplementary Fig. 1, respectively) present an induced charge density of $-\sigma$. Given that the net charges on both electrodes should be zero in open circuit condition, the induced charge density on overlapped regions (regions 2 and 3) can be expressed as

Overlapped part on electrode 1 (region 2): $\rho = -\sigma \frac{\theta_0}{\theta_0 - \theta}$ (1)

Overlapped part on electrode 2 (region 3): $\rho = -\sigma \frac{\theta_0}{\theta_0 - \theta}$ (2)

Where θ_0 refers to the central angle of the rotator unit, α refers to rotation angle away from the initial position between 0 and θ_0 .

Based on the law of charge conservation, the charge density on different regions of the rotator can be expressed as

Region 2 of the rotator: $\rho = \sigma + \sigma \frac{\theta_0}{\theta_0 - \theta}$ (3)

Region 3 of the rotator: $\rho = \sigma + \sigma \frac{\theta_0}{\theta_0 - \theta}$ (4)

Using the charge density shown in Supplementary S1 and Gauss Theorem, the electric field within the dielectric layer for region 2 and 3 can be respectively given as,

$$E_{Region2} = -\frac{\sigma}{\varepsilon_0 \varepsilon_r} \cdot \frac{\theta}{\theta_0 - \theta} \quad (5)$$

$$E_{Region3} = -\frac{\sigma}{\varepsilon_0 \varepsilon_r} \cdot \frac{\theta_0 - \theta}{\theta} \quad (6)$$

where ε_r is the relative permittivity of dielectric layer.

Then the potential difference between the rotator and electrode 1(E_1) as well as the potential difference between the rotator and electrode 2 (E_2) can be respectively calculated as,

$$V_{Rotator} - V_{E1} = \frac{d\cdot\sigma}{\varepsilon_0\varepsilon_r}\cdot\frac{\theta}{\theta_0-\theta} \tag{7}$$

$$V_{Rotator} - V_{E2} = \frac{d\cdot\sigma}{\varepsilon_0\varepsilon_r}\cdot\frac{\theta_0-\theta}{\theta} \tag{8}$$

where d is the thickness of the dielectric layer.

Since the rotator made of metal is an equipotential body, the potential difference between the two electrodes (i.e. open-circuit voltage) can be theoretically expressed as

$$V_{OC}(\theta) = V_{E1} - V_{E2} = \frac{d\cdot\sigma}{\varepsilon_0\varepsilon_r}\cdot\frac{\theta}{\theta_0-\theta} - \frac{d\cdot\sigma}{\varepsilon_0\varepsilon_r}\cdot\frac{\theta_0-\theta}{\theta} \tag{9}$$

which is,

$$V_{OC}(\theta) = U_{E1} - U_{E2} = \frac{d\cdot\sigma}{\varepsilon_0\varepsilon_r}\left(\frac{\theta}{\theta_0-\theta} - \frac{\theta_0-\theta}{\theta}\right) \tag{10}$$

However, the above equation S (10) is not applicable when approaches either 0 or θ_0 . When θ approaches 0, the V_{OC} obtained by this equation goes to positive infinity. This is because when α has a very small value, the rotator only has a very small overlapped area (region 3 in Supplementary Fig. 1) with E_2. In this case, the assumption of parallel-plate capacitor does not hold any more. Therefore, deviation occurs. Similarly, when α approaches θ_0 , the overlapped area between the rotator and E_1 (region 2 in Supplementary Fig. 1) is so small that the basic assumption of parallel-plate capacitor also no longer holds, resulting in negative infinite value of V_{OC} from equation S (10). Therefore, equation S (10) is only used to illustrate the changing trend of the V_{OC} when the rotator spins. In order to calculate the V_{OC} at the initial and final positions, the following derivation based on electrostatics is used.

At region 1 on the left (Supplementary Fig. 2), the net triboelectric charge at the contact interface is σ , while the net triboelectric charge is – σ at region 2 on the right. Based on the model of infinitely large plane with uniform charging, the electric potential of E_1 and E_2 with an infinitely far position as a zero-potential reference point can be respectively calculated by

$$V_{\mathrm{E1}} = \frac{d \cdot \sigma}{\varepsilon_0 \varepsilon_r} \quad (11)$$

$$V_{\mathrm{E2}} = -\frac{d \cdot \sigma}{\varepsilon_0 \varepsilon_r} \quad (12)$$

Therefore, the V_{OC} at the initial state is

$$V_{OC(inirial)} = V_{\mathrm{E1}} - V_{\mathrm{E2}} = \frac{2d \cdot \sigma}{\varepsilon_0 \varepsilon_r} \quad (13)$$

Based on the same reasoning, the $\mathrm{V_{OC}}$ at the final state is

$$V_{OC(final)} = V_{E1} - V_{E2} = -\frac{2d \cdot \sigma}{\varepsilon_0 \varepsilon_r} \quad (14)$$

Consequently, the peak-to-peak value of the $\mathrm{V_{OC}}$ is

$$V_{p-p} = V_{E1} - V_{E2} = \frac{4d \cdot \sigma}{\varepsilon_0 \varepsilon_r} \quad (15)$$

Supplementary Note S3│Theoretical analysis of operating process in short-current condition.

Based on the model of volume-changing capacitors, we can assume a voltage ($V_{E1\,E2}$)-charge ($Q_{E1\,E2}$) relationship between E_1 and E_2 as follows,

$$V_{E1\,E2} = -\frac{1}{C_{E1\,E2}} \times Q_{E1\,E2} + V_{OC} \quad (16)$$

where $C_{E1\,E2}$ is the capacitance between E_1 and E_2.

$C_{E1\,E2}$ can be treated as a series connection of two capacitors, which are the capacitor formed by the rotator and E_1 and the capacitor formed by the rotator and E_2:

$$C_{\mathrm{E1\,E2}} = \frac{1}{\frac{1}{C_{Rotator-E1}}+\frac{1}{C_{Rotator-E2}}} \tag{17}$$

$$C_{Rotator-E1} = \frac{\varepsilon_0\varepsilon_r\, P(\theta_0-\theta)}{d} \tag{18}$$

$$C_{Rotator-E2} = \frac{\varepsilon_0\varepsilon_r\, P(\theta)}{d} \tag{19}$$

where $P(\theta_0 - \theta)$ is the overlapped area between the rotator and E_1, and $P(\theta)$ is the overlapped area between the rotator and E_2.

Finally, we can get

$$V_{E1E2} = -\left[\frac{d\cdot\theta_0}{\varepsilon_0\varepsilon_r\cdot\theta}\cdot\frac{360^o}{\theta_0-\theta}\cdot\frac{1}{\pi(r_2^2-r_1^2)}\right]\times Q_{E1\,E2} + \frac{d\cdot\sigma}{\varepsilon_0\varepsilon_r}\left(\frac{\theta}{\theta_0-\theta}-\frac{\theta_0-\theta}{\theta}\right) \tag{20}$$

where r_2 is the outer radius of the rotator, and r_1 is the inner radius of the rotator. In short circuit condition, $V_{E1,E2} = 0$. Therefore, charge transferred between the two electrodes in short circuit condition is,

$$Q_{E1\,E2} = \frac{2\theta_0-\theta}{360^o}\cdot\sigma\cdot\pi(r_2^2-r_1^2) \tag{21}$$

By submitting $\theta = 0$ and $\theta_0 = \theta$ into equation (20), we can obtain the total charge that transport as the rotator spins from $\theta = 0$ to $\theta_0 = \theta$ by the following equation

$$Q = \frac{2\theta_0}{360^o}\cdot\sigma\cdot\pi(r_2^2-r_1^2) \tag{22}$$

The Voltage Equation Models

To describe a system with two voltage sources working inversely, where the first source generates voltage for 2 seconds and then stops, allowing the second source to start generating voltage immediately afterward for 2 seconds, we can model the behavior using a piecewise function over time. Let's define the following:

- V_{U1}(t): Voltage generated by the first source.
- V_{U2}(t): Voltage generated by the second source.
- (t): Time in seconds.

The system repeats the cycle every 4 seconds, with the first source working for 2 seconds and the second source working for the next 2 seconds. The total voltage $Vs_{oc\,p-p}$(t): is the sum of the two Voc peak to peak for each Unit (VU1, VU2), but since only one source works at a time, we can express the voltage as follows:

$$Vs_{oc\,p-p}(t) = \begin{cases} V_{U1}(t), & 0 \leq t_p < \frac{t_p}{2} \\ V_{U2}(t), & \frac{t_p}{2} \leq t_p < t_p \end{cases} \tag{23}$$

where t_p is the time modulated by 4 seconds (so the behavior repeats every 4 seconds). If t_p goes beyond 4 seconds, it will reset, maintaining the cyclic behavior:

Voltage for Source V_{U1} (t):

Source 1 operates for the first 2 seconds. During this period, the voltage generated by the source will be:

$$V_{U1}(\mathrm{t}) = \frac{4d \cdot \sigma}{\varepsilon_0 \varepsilon_r} \tag{24}$$

For 0 $\leq t_p < \frac{1}{2} t_p$, V_{U1} (t) remains constant because the variables d, σ, ε_0 and ε_r are constant in the given equation.

Voltage for Source V_{U2} (t):

Source 2 operates immediately after Source 1, from $t = \frac{1}{2}t_p$ seconds to $t_p = 4$ seconds. For this source, the voltage will also be based on the same equation:

$$V_{U2}(\mathrm{t}) = \frac{4d\cdot\sigma}{\varepsilon_0\varepsilon_r} \tag{25}$$

For $\frac{1}{2}t_p \leq t < t_p$ also remains constant, similar to V_{U1}(t)

Supplementary Note S4 | Gear configuration and energy transfer, optimization of energy transfer and calculations of speeds and ratios.

Gear configuration and energy transfer

Our gear system utilizes a multi-stage configuration to efficiently convert linear motion from the rack gear into rotational motion at the output shaft. The specific gear ratios were carefully selected to balance speed and torque, ensuring optimal performance under different speeds and forces.

The system begins with an initial gear reduction from the rack gear to Gear 1. This stage reduces the high-speed linear motion of the rack and increases torque, providing a mechanical advantage. The intermediate gear stages, including Gears 2 and 3, further refine the speed and torque output. The final stage, a gear amplification from Gear 4 to Gear 5, significantly increases the speed of the output while reducing torque, making the system suitable for applications requiring rapid motion transfer.

Optimization of Energy Transfer

To minimize energy loss and ensure efficiency, the gear system incorporates the following measures:

Material Selection: Nylon was chosen for the gears due to its self-lubricating properties, lightweight design, and low noise generation. Nylon also offers advantages such as corrosion resistance and ease of manufacturing, making it ideal for low-load and low-speed applications.

Calculations of Speeds and Ratios

We have calculated the gear speeds and ratios to further illustrate the energy transfer process. The gear system includes a rack gear and five rotational gears with the following dimensions as shown in Supplementary Figure S5:

Rack Gear: Length = 8 cm, Teeth = 26, Linear Speed = 2 cm/s

Gear 1: Teeth = 30, **Pitch Diameter** = 32 cm

Gear 2: Teeth = 60, **Pitch Diameter** = 62 cm

Gear 3: Teeth = 30, **Pitch Diameter** = 32 cm

Gear 4: Teeth = 60, **Pitch Diameter** = 62 cm

Gear 5: Teeth = 15, **Pitch Diameter** = 17 cm

Step-by-Step Calculations

Rack to Gear 1: Gear Ratio: 26/30

Speed of Gear 1: $\frac{26}{30} \times 2$ =1.73 cm/s

Gear 2 (Same Shaft as Gear 1):

Rotational Speed = 1.73 cm/s

Gear 3 (Driven by Gear 2):

Gear Ratio: 2

Speed: 1.73 cm/s ×2=3.46 cm/s

Gear 4 (Same Shaft as Gear 3):

Gear 5 (Driven by Gear 4):

Gear Ratio: 4

Speed: 3.46×4=13.84 cm/s

Supplementary Table S1｜Show the details of the gears speed.

**Input speed (cm/s)**	**Gear 1,2 (cm/s)**	**Gear 3,4 (cm/s)**	**Gear 5 (cm/s)**
**1.44**	1.25	2.5	10
**2**	1.73	3.46	13.84
**2.66**	2.3	4.61	18.43
**3.5**	3.03	6.06	24.25
**4**	3.47	6.93	27.72
**5.34**	4.63	9.26	37.05
**6.4**	5.55	11.1	44.4
**8**	6.93	13.86	55.44

Supplementary Table S2 | The comparison of power density and volume of previously reported TENGs for energy harvesting.

	Year	Name	Working Mode	Application					
				**Power (mW)**	**Power Density ($W/m^3 \cdot Hz$)**	**Optimal Resistance**	**Volume (cm^3)**	**Ref.**
1	2024	DHLR-TENG	Sliding Mode	**Peak: 33.27 (Motor, 1Hz)**	**171.616**	**9 MΩ**	**193.865**	This work
2	2023	SR-TENG	Rolling mode	**Peak: 16** (Motor, 2 Hz)	**Peak: 26.435** (Motor, 2 Hz)	**300 MΩ** (Motor, 2 Hz)	**302.63**	[1]
3	2023	FE-TENG	Rolling mode	**N/A**	**Peak: 5.6** (Motor, 1.25 Hz)	**1000 MΩ** (Motor, 1.25 Hz)	**N/A**	[2]
4	2019	Self-assembly TENG	Rolling mode	**Peak: 8.75** (Motor, 1.67 Hz)	**Peak: 19.55** (Motor, 1.67 Hz)	**1000 MΩ** (Motor, 1.67 Hz)	**268**	[3]
5	2023	Chiral Network of Triboelectric Nanogenerator	Rolling mode	**Peak: 7.88** (motor, 1.5 Hz)	**Peak: 19.61** (motor, 1.5 Hz)	**1000 MΩ** (Motor, 1 Hz)	**267.95**	[4]
6	2021	Spherical TENG	Rolling mode	**Peak: 10.7** (Motor, 5.9 Hz)	**Peak: 3.47** (Motor, 5.9 Hz)	**300 MΩ** (Motor, 5.9 Hz)	**527**	[5]
7	2019	T-TENG	Rolling mode	**Peak: 0.143** (Motor, 1.2 Hz)	**Peak: 1.03** (Motor, 1.2 Hz)	**2000 MΩ** (Motor, 1.2 Hz)	**1390**	[6]
8	2021	S-TENG	Rolling mode	**Peak: 25.22** (Motor, 2 Hz)	**Peak: 17.325** (Motor, 2 Hz)	**500 MΩ** (Motor, 2 Hz)	**727.85**	[7]

9	2022	S-TENG	Rolling mode	**N/A**	**Peak: 24.535** (Motor, 2 Hz)	**300 MΩ** (Motor, 2 Hz)	**N/A**	[8]
10	2021	WT-TENG	Sliding Mode	**N/A**	**Peak: 13.1** (Motor, 1 Hz)	**Peak: 10 GΩ** (Motor, 1 Hz)	**5.2**	[9]
11	2020	CS-TENG	Contact-Separation	**Peak: 126.8** (Motor, 1 Hz)	**Peak: 17.81** (Motor, 1 Hz)	**517 kΩ** (Motor, 0.7 Hz)	**488.79**	[10]
12	2019	OB-TENG	Contact-Separation	**Peak: 38.7** (Motor, 1 Hz)	**Peak: 9.675** (Motor, 1 Hz)	**13.8 MΩ** (Motor, 1 Hz)	**4000**	[11]
13	2023	DE-TENG	Sliding Mode	**Peak: 0.85** (Motor, 0.5 Hz)	**Peak: 61.9** (Motor, 0.5 Hz)	**500 MΩ**	**27.3**	[12]
14	2023	CS-TENG	Sliding Mode	**Peak: 14.36** (motor, 1.75 Hz)	**Peak: 19.71** (motor, 1.75 Hz)	**560 kΩ** (Motor, 1.75 Hz)	**416**	[13]
15	2019	TD-TENG	Sliding Mode	**Peak: 29.4** (Motor, 1 Hz)	**Peak: 30.21** (Motor, 1 Hz)	**1 MΩ** (Motor, 1 Hz)	**973.3**	[14]
16	2023	WLM-TENG	Sliding Mode	**Peak: 50** (Wave, 1 Hz)	**Peak: 14.1** (Wave, 1 Hz)	**200 kΩ** (Wave, 1 Hz)	**3546.09**	[15]

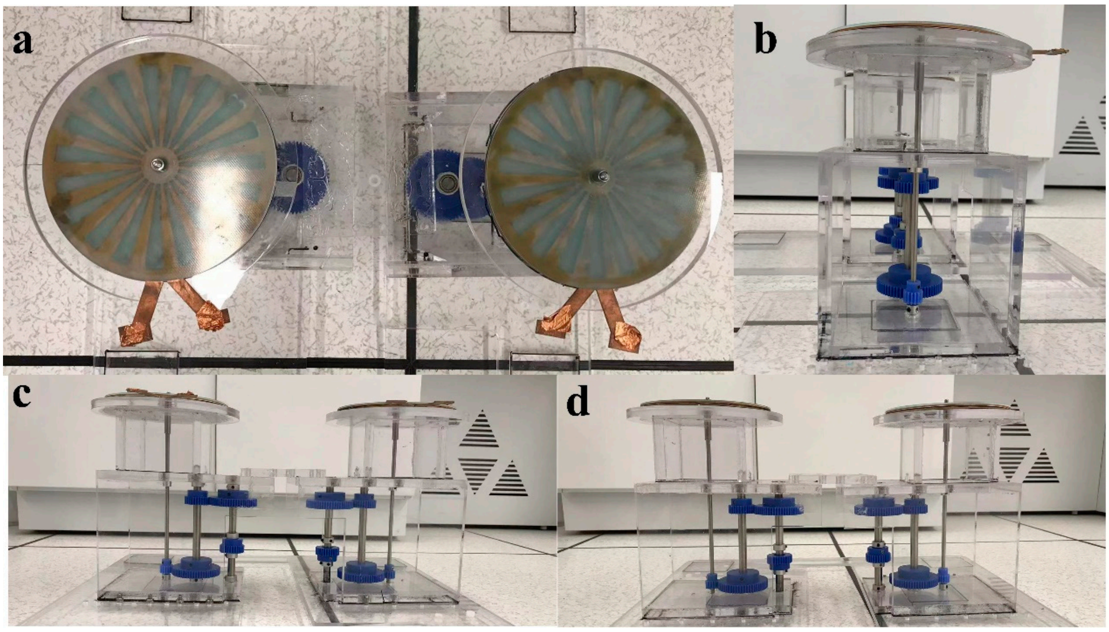

Supplementary Figure S12 | The pictorial images of (a) top, (b) right, (c) back, and (d) front side of the device.

References

[1] Y. Duan, H. Xu, S. Liu, P. Chen, X. Wang, L. Xu, T. Jiang, Z.L. Wang, Scalable rolling-structured triboelectric nanogenerator with high power density for water wave energy harvesting toward marine environmental monitoring, Nano Research 16 (2023) 11646-11652.

[2] Z. Jing, J. Zhang, J. Wang, M. Zhu, X. Wang, T. Cheng, J. Zhu, Z.L. Wang, 3D fully-enclosed triboelectric nanogenerator with bionic fish-like structure for harvesting hydrokinetic energy, Nano Research 15 (2022) 5098-5104.

[3] X. Yang, L. Xu, P. Lin, W. Zhong, Y. Bai, J. Luo, J. Chen, Z.L. Wang, Macroscopic self-assembly network of encapsulated high-performance triboelectric nanogenerators for water wave energy harvesting, Nano Energy 60 (2019) 404-412.

[4] X. Li, L. Xu, P. Lin, X. Yang, H. Wang, H. Qin, Z.L. Wang, Three-dimensional chiral networks of triboelectric nanogenerators inspired by metamaterial's structure, Energy & Environmental Science 16 (2023) 3040-3052.

[5] Z. Yuan, C. Wang, J. Xi, X. Han, J. Li, S.-T. Han, W. Gao, C. Pan, Spherical triboelectric nanogenerator with dense point contacts for harvesting multidirectional water wave and vibration energy, ACS Energy Letters 6 (2021) 2809-2816.

[6] M. Xu, T. Zhao, C. Wang, S.L. Zhang, Z. Li, X. Pan, Z.L. Wang, High power density tower-like triboelectric nanogenerator for harvesting arbitrary directional water wave energy, ACS nano 13 (2019) 1932-1939.

[7] H. Wang, Z. Fan, T. Zhao, J. Dong, S. Wang, Y. Wang, X. Xiao, C. Liu, X. Pan, Y. Zhao, Sandwich-like triboelectric nanogenerators integrated self-powered buoy for navigation safety, Nano Energy 84 (2021) 105920.

[8] H. Wang, C. Zhu, W. Wang, R. Xu, P. Chen, T. Du, T. Xue, Z. Wang, M. Xu, A stackable triboelectric nanogenerator for wave-driven marine buoys, Nanomaterials 12 (2022) 594.

[9] H. Wu, Z. Wang, Y. Zi, Multi-mode water-tube-based triboelectric nanogenerator designed for low-frequency energy harvesting with ultrahigh volumetric charge density, Advanced Energy Materials 11 (2021) 2100038.

[10] H. Wang, L. Xu, Y. Bai, Z.L. Wang, Pumping up the charge density of a triboelectric nanogenerator by charge-shuttling, Nature Communications 11 (2020) 4203.

[11] W. Zhong, L. Xu, X. Yang, W. Tang, J. Shao, B. Chen, Z.L. Wang, Open-book-like triboelectric nanogenerators based on low-frequency roll–swing oscillators for wave energy harvesting, Nanoscale 11 (2019) 7199-7208.

[12] X. Liang, S. Liu, S. Lin, H. Yang, T. Jiang, Z.L. Wang, Liquid–solid triboelectric nanogenerator arrays based on dynamic electric-double-layer for harvesting water wave energy, Advanced Energy Materials 13 (2023) 2300571.

[13] H. Qiu, H. Wang, L. Xu, M. Zheng, Z.L. Wang, Brownian motor inspired monodirectional continuous spinning triboelectric nanogenerators for extracting energy from irregular gentle water waves, Energy & Environmental Science 16 (2023) 473-483.

[14] Y. Bai, L. Xu, C. He, L. Zhu, X. Yang, T. Jiang, J. Nie, W. Zhong, Z.L. Wang, High-performance triboelectric nanogenerators for self-powered, in-situ and real-time water quality mapping, Nano Energy 66 (2019) 104117.

[15] J. Han, Y. Liu, Y. Feng, T. Jiang, Z.L. Wang, Achieving a large driving force on triboelectric nanogenerator by wave-driven linkage mechanism for harvesting blue

energy toward marine environment monitoring, Advanced Energy Materials 13 (2023) 2203219.
